# An exploration of the dynamic longitudinal relationship between mental health and alcohol consumption: a prospective cohort study

**DOI:** 10.1186/1741-7015-12-91

**Published:** 2014-06-03

**Authors:** Steven Bell, Annie Britton

**Affiliations:** 1 Research Department of Epidemiology and Public Health, University College London, 1-19 Torrington Place, London, UK

**Keywords:** Alcohol, Mental health, Longitudinal, Reciprocal, Self-medication, Temporality

## Abstract

**Background:**

Despite intense investigation, the temporal sequence between alcohol consumption and mental health remains unclear. This study explored the relationship between alcohol consumption and mental health over multiple occasions, and compared a series of competing theoretical models to determine which best reflected the association between the two.

**Methods:**

Data from phases 5 (1997 to 1999), 7 (2002 to 2004), and 9 (2007 to 2009) of the Whitehall II prospective cohort study were used, providing approximately 10 years of follow-up for 6,330 participants (73% men; mean ± SD age 55.8 ± 6.0 years). Mental health was assessed using the Short Form (SF)-36 mental health component score. Alcohol consumption was defined as the number of UK units of alcohol drunk per week. Four dynamic latent change score models were compared: 1) a baseline model in which alcohol consumption and mental health trajectories did not influence each other, 2) and model in which alcohol consumption influenced changes in mental health but mental health exerted no effect on changes in drinking and 3) *vice versa*, and (4) a reciprocal model in which both variables influenced changes in each other.

**Results:**

The third model, in which mental health influenced changes in alcohol consumption but not *vice versa*, was the best fit. In this model, the effect of previous mental health on upcoming change in alcohol consumption was negative (γ = -0.31, 95% CI -0.52 to -0.10), meaning that those with better mental health tended to make greater reductions (or shallower increases) in their drinking between occasions.

**Conclusions:**

Mental health appears to be the leading indicator of change in the dynamic longitudinal relationship between mental health and weekly alcohol consumption in this sample of middle-aged adults. In addition to fuelling increases in alcohol consumption among low-level consumers, poor mental health may also be a maintaining factor for heavy alcohol consumption. Future work should seek to examine whether there are critical levels of alcohol intake at which different dynamic relationships begin to emerge between alcohol-related measures and mental health.

## Background

Alcohol consumption [1,2] and mental health [3-5] are two of the biggest public health issues facing modern society. The relationship between alcohol consumption and mental health has been documented extensively [6-13], and there have been several ways proposed as to how the relationship may operate [14]. Plausible biological mechanisms for hazardous alcohol consumption leading to depression include alcohol reducing white and gray matter volumes, as well as influencing neurotransmitter functioning [15]. Changes in white and gray matter volume [16,17], and the microstructure of nerve fibers [18] are thought to be related to major depression, while the dysregulation of GABAergic [19,20], dopaminergic [21], and serotonergic [22,23] systems are widely supported hypotheses in the etiology of depression. Hazardous alcohol consumption can create tension in home/work environments [24], which may exacerbate marital disputes [25], lead to job loss [26], and result in other stressful scenarios, which in turn can lead to poor mental health. Clinical studies have also demonstrated that individuals treated for alcohol dependence show marked decreases in symptoms of poor mental health following a period of abstinence [27] suggesting that alcohol may be the primary causal factor. Theoretical explanations for poor mental health influencing alcohol intake include the use of alcohol as a coping mechanism for tension and depression/anxiety [28-32]. A meta-analysis of literature around the 'self-medication' hypothesis found that depression can lead to increased alcohol consumption, and then progression to alcohol-use disorders [33].

The current evidence base is mixed; some authors have found that the driving force is alcohol, while others have concluded that it is mental health. It is also hypothesized that dynamic feedback cycles contribute to the escalation of alcohol consumption and worsening mental health [34]; that is, people may become depressed or anxious and turn to alcohol, which causes them to become more depressed or anxious, which eventually fuels further drinking, or the reverse may occur, with drinking leading to symptoms of anxiety or depression, which encourage further drinking. Yet, few studies have empirically tested this theory and those that have are limited by the methods used to try to capture the dynamic interplay between both variables over time [35] (e.g. not taking into account repeated measures of both variables in the same model). Using repeated longitudinal data on both alcohol consumption and mental health symptoms would allow for hypotheses of leading indicators of change (that is, alcohol consumption driving changes in mental health, or *vice versa*) as well as reciprocal relationships to be tested. Understanding the temporal sequence of the relationship between the two processes over time is important to public health because it will allow for interventions/prevention strategies to be tailored more effectively.

Furthermore, studies on alcohol consumption and mental health have mostly been concerned with the comorbid relationship between clinical disorders [10], not on sub-syndrome symptoms of mental health (that is. pre-clinical manifestations), which make up a greater proportion of the overall burden of mental health [36] or on the drinking habits of the general population. Previous studies have also tended to focus on the transition or maintenance of a clinical state or binary 'heavy drinker' or 'symptoms of mental health problems' [37,9]. As the trajectory from disease free to clinical disorder is not as simple as moving from one state to another but is instead characterized by an escalation of symptoms and behaviors, it might be argued that other studies have failed to effectively capture the 'true' longitudinal relationship between mental health and alcohol consumption. Knowing how the relationship between alcohol consumption and mental health operates prior to the development of clinical disorders would allow for primary prevention strategies to be targeted more effectively.

The purpose of this study was therefore to address these limitations by exploring the longitudinal relationship between alcohol consumption and mental health symptoms jointly over multiple occasions in a general population setting, and to compare several competing theoretical models to determine which best reflected the association between these two factors.

## Materials and methods

### The Whitehall II study

The Whitehall II prospective cohort study started with a sample of 10,308 British civil servants (6,895 men and 3,413 women), who were aged 34 to 56 years at entry into the study (1985 to 88) [38]. The current investigation uses of data from three clinical phases: 5 (1997 to 1999; referred to hereafter as 'baseline'), 7 (2002 to 2004) and 9 (2007 to 2009). At baseline, the total number of eligible participants was 7,870. Those who had not consumed alcohol in the year before baseline and additionally those with missing values for either alcohol consumption or mental health variables at baseline were excluded from the analytic sample (n = 548 and n = 1,036 respectively; categories were not mutually exclusive). This provided approximately 10 years of follow-up information for 6,330 participants who had consumed alcohol in the year before baseline.

The University College London Medical School Committee on the ethics of human research approved the Whitehall II study.

### Assessment of alcohol consumption

Participants were asked to report the number of drinks they had consumed in the previous week, quoting separately for beer/cider (pints), wine (glasses), and spirits (measures). Drinks were converted into UK units of alcohol (one unit is equivalent to 8 g of ethanol) using a conservative estimate of one UK unit for each measure of spirits and glass of wine, and two UK units for each pint of beer. These converted measurements were then summed to define the total weekly number of UK units consumed.

### Assessment of mental health

Mental health (combining symptoms of depression and anxiety) was assessed using the mental health component score [39,40] (MCS) of the Short Form (SF)-36 questionnaire [41]. The SF-36 refers to symptoms experienced in the previous 4 weeks. The MCS has been validated using UK data sources [42], and reliability estimates typically exceed values of 0.90 for Cronbach’s α [39]. The MCS uses a scale of 0 to 100, with higher scores indicating better functioning.

### Covariates

Adjustment was made for several baseline covariates, including age, sex, ethnicity, socioeconomic status, marital status, highest educational qualification, economic activity, social network [43], smoking status, level of physical activity [44]. and use of anti-depressant medication. Problematic alcohol consumption (defined by the CAGE questionnaire [45]) was used to adjust for the possibility that problem drinking may be driving any observed relationship [46].

Poor physical health could influence both alcohol consumption [47,48] and mental health [49] trajectories. Therefore, general physical health was accounted for by adjusting for several chronic conditions. A combination of self-report and validated clinical health events [50] were included, such as self-reported long-standing physical illness and belonging to the lowest sex-specific SF-36 physical health component quartile [51], as well as known diabetes mellitus, coronary heart disease (CHD), stroke, transient ischemic attack (TIA), total serum cholesterol, systolic and diastolic blood pressure, resting heart rate over 80 beats/min [52], and body mass index (BMI).

### Statistical analysis

Bivariate latent change score (LCS) models [35,53-57] were used to explore the dominant temporal sequence in the longitudinal relationship between alcohol consumption and mental health symptoms. LCS models are an extension of standard growth curve models [58] (also referred to as random effects models) and acknowledge that repeated measures on the same individual are correlated. A general overview of the underlying assumptions and specification of LCS models are presented (see Additional file 1), but a comprehensive outline of the mathematical and statistical properties [55,57], as well as a comparison of LCS models with other multivariate longitudinal models [35] can be found elsewhere.

Briefly, there are three primary parameters of interest: 1) the slope parameter (α), which refers to the additive sum of changes during follow-up; 2) the autoproportional parameter (β), which refers to the lagged effect of a variable on an upcoming change in itself (self-feedback); and 3) the coupling parameter (γ), which describes the lagged effect of one variable on the upcoming change in the alternate variable.

Both the intercept and the slope were fitted as random effects, allowing for them to vary between individuals. Intercepts and slopes (as well as their random effects) were correlated (ρ) both within a single process (for example, the alcohol consumption intercept with the alcohol slope) and between processes (for example, the mental health intercept with the alcohol slope). Intercepts and slopes were estimated conditional on the baseline covariates described above.

Four separate models were estimated: 1) **no coupling (baseline) model**; 2) **alcohol consumption producing change in mental health model**; 3) **mental health producing** c**hange in alcohol consumption model; and** 4) **dynamic/reciprocal change model**. Nested models were compared to determine the best-fitting model (that is, to justify the inclusion of either or both coupling parameters over a baseline model that did not contain them) using a χ^2^ difference test.

The relative fit of the hypothesized models compared with the observed data was assessed using the Tucker–Lewis index (TLI), the comparative fit index (CFI), and the root mean squared error of approximation (RMSEA). Cut-off values close to 0.95 were used to determine a good fit for TLI and CFI, while a cut-off value close to 0.06 was used for RMSEA [59].

Models were estimated in Mplus v6.12 [60] using the full information maximum likelihood (FIML) estimator [61]. An α level of 0.05 was considered statistically significant for all analyses.

## Results

### Sample composition

**Figure 1:**
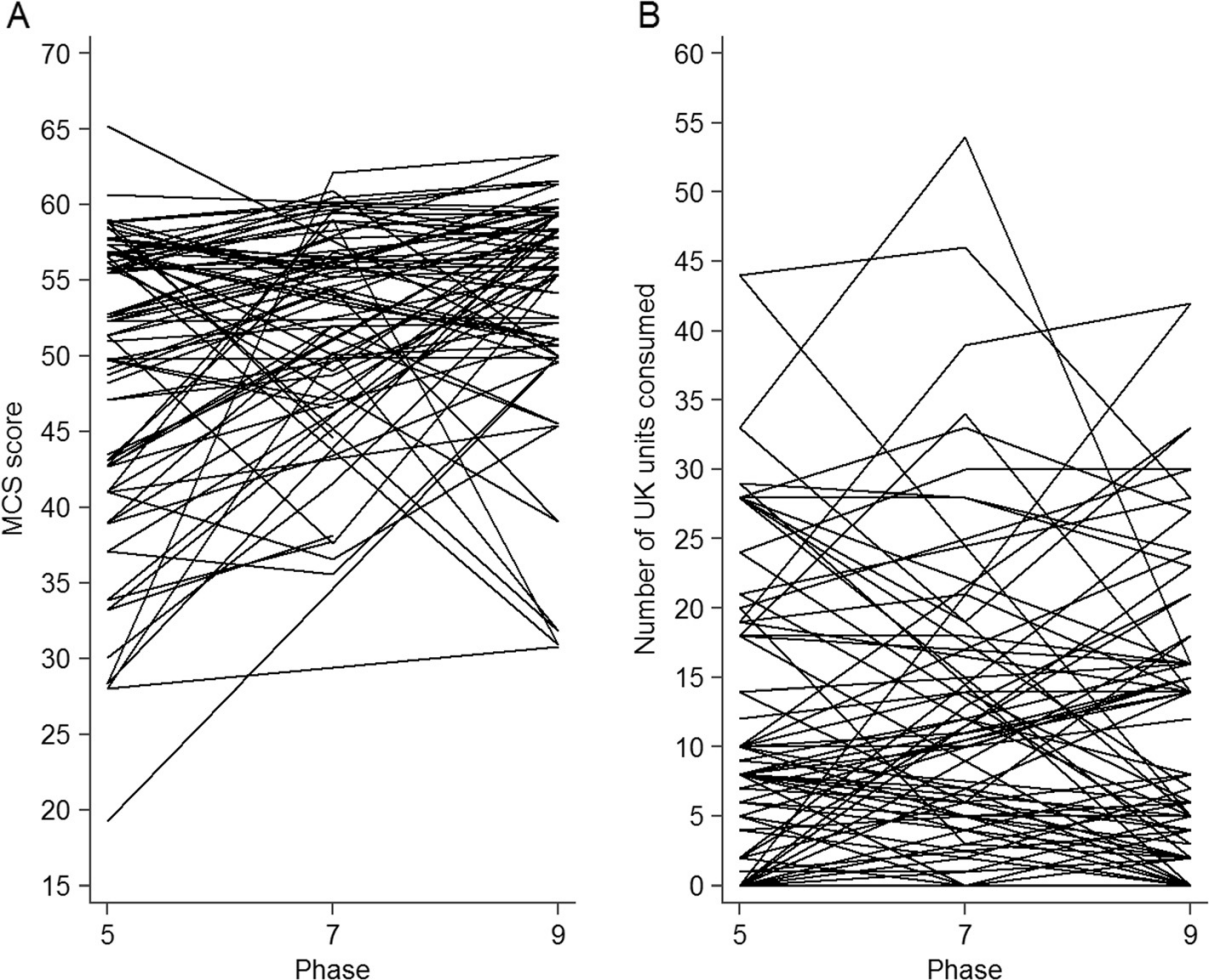
Spaghetti plots of the observed longitudinal trajectories of mental health symptoms (panel A) and alcohol consumption (panel B) in a random sample (n = 50) of participants in the Whitehall II study.

Table 1 displays descriptive statistics for the complete analytic sample (based on observed information only). The mean age of participants was 56 years, over 70% were male, and approximately 6% were non-white. Most were of high to intermediate socioeconomic status, almost four-fifths were married or cohabiting, over 60% had post-secondary or university level qualifications, and around 65 were economically active. Almost 11% were current smokers, 11% were identified as problem drinkers, and 70% were physically active. In terms of physical health, 6% of the sample had known CHD, 4% had known diabetes mellitus, 0.5% had experienced a stroke, 0.7% had experienced a TIA, almost 3% were currently being prescribed anti-depressant medication, and approximately half reported a long-standing illness.At baseline, participants consumed on average 14.5 UK units of alcohol per week, and this figure had reduced to 11 UK units by the end of follow-up. Mental health scores started at an average of 51 and increased to 54 (a random selection of observed (A) mental health and (B) alcohol consumption trajectories are displayed in Figure 1).

**Table 1:** Sample characteristics

	**n**	**% or mean ± SD**
MCS		
Phase 5	6,330	51.1 ± 9.4
Phase 7	5,436	52.4 ± 8.8
Phase 9	5,195	53.8 ± 8.0
UK units of alcohol		
Phase 5	6,330	14.6 ± 15.2
Phase 7	5,508	13.0 ± 13.0
Phase 9	5,215	11.1 ± 11.3
Age, years	6,330	55.8 ± 6.0
Sex		
Male	4,594	72.6
Female	1,736	27.4
Total	6,330	
Ethnicity		
White	5,966	94.25
Non-white	364	5.75
Total	6,330	
SES		
High	2,852	45.3
Intermediate	2,731	43.4
Low	713	11.3
Total	6,296	
Marital status		
Married/cohabiting	4,861	79.6
Other	1,248	20.4
Total	6,109	
Education		
University	2,176	36.4
Post-secondary	1,648	27.5
Secondary	1,558	26.0
No qualifications	604	10.1
Total	5,986	
Economic activity		
Active	4,123	65.2
Inactive	2,203	34.8
Total	6,326	
Current smoker		
No	5,539	89.4
Yes	654	10.6
Total	6,193	
Problem drinking (CAGE case)		
No	5,531	89.0
Yes	684	11.0
Total	6,215	
Physical activity		
Active	3,405	54.16
Moderately active	1,057	16.78
Inactive	1,837	29.16
Total	6,299	
Network score	6,053	7.3 ± 3.0
CHD		
No	5,948	94.0
Yes	382	6.0
Total	6,330	
Known diabetes		
No	6,076	96.0
Yes	254	4.0
Total	6,330	
Anti-depressant medication		
No	6,149	97.3
Yes	171	2.7
Total	6,320	
Poor self-reported physical health		
No	4,821	76.2
Yes	1,509	23.8
Total	6,330	
Long-standing illness		
No	3,248	51.4
Yes	3,075	48.6
Total	6,323	
Stroke		
No	6,301	99.5
Yes	29	0.5
Total	6,330	
TIA		
No	6,287	99.3
Yes	43	0.7
Total	6,330	
Resting heart rate > 80 bpm		
No	4,969	88.15
Yes	668	11.85
Total	5,637	
BMI	4,916	26.1 ± 3.9
Serum cholesterol, mmol/l	5,622	5.9 ± 1.1
Blood pressure, mmHg		
Systolic	5,669	123.1 ± 16.4
Diastolic	5,669	77.6 ± 10.6

BMI, body mass index; bpm, beats per minute; CHD, coronary heart disease; MCS, mental health component score; SES, socioeconomic status; TIA, transient ischemic attack.

### Regression estimates

Indices related to model fit and statistics concerning model comparison are shown in Table 2. All models specified were well fitting according to commonly accepted thresholds of model fit as outlined above [59]. Detailed estimates for the best-fitting model are presented in Table 3,while only the fixed effect parameters are presented for other models specified in Table 4 (random effects for these models can be found in Additional file 2: Table S2A).

**Table 2:** Model fit indices and comparison of LCS models for total weekly alcohol consumption and mental health in the Whitehall II study

	**Baseline**	**Alcohol → ΔMCS**	**MCS → Δalcohol**	**Reciprocal**
Age and sex adjusted				
Fit statistics				
Log likelihood	-146158.161	-146158.041	-146155.151	-146154.760
χ^2^ (df)	274.233 (12)	273.995 (11)	268.214 (11)	267.432 (10)
RMSEA	0.059	0.061	0.061	0.064
AIC	292380.321	292382.083	292376.302	292377.520
SSA BIC	292494.731	292500.068	292494.288	292499.081
CFI	0.982	0.982	0.982	0.982
TLI	0.959	0.955	0.956	0.952
Model comparison (difference in χ^2^ fit (df))				
Versus baseline	–	0.238 (1), *P* = 0.63	6.019 (1), *P* = 0.01	6.801 (2), *P* = 0.03
Versus previous best	–	–	–	0.782 (1), *P* = 0.38
Fully adjusted				
Fit statistics				
Log likelihood	-243989.314	-243989.108	-243985.355	-243984.851
χ^2^ (df)	328.239 (54)	327.827 (53)	320.320 (53)	319.312 (52)
RMSEA	0.028	0.029	0.028	0.028
AIC	488798.629	488800.217	488792.710	488793.702
SSA BIC	490264.509	490269.672	490262.165	490266.732
CFI	0.983	0.983	0.984	0.984
TLI	0.952	0.951	0.953	0.952
Model comparison (difference in χ^2^ fit (df))				
Versus baseline	–	0.412 (1), *P* = 0.52	7.919 (1), *P* < 0.01	8.927 (2), *P* < 0.01
Versus previous best	–	–	–	1.008 (1), *P* = 0.32

AIC, Akaike information criterion; CFI, comparative fit index; df, degrees of freedom; LCS, latent change score; MCS, mental health component score; RMSEA, root mean square error of approximation; SSA BIC, sample size adjusted Bayesian information criterion; TLI, Tucker-Lewis index.

**Table 3:** **Parameter estimates (95% confidence intervals) for the best-fitting LCS model of weekly alcohol consumption and mental health symptoms in the Whitehall II study**^**a **^**(MCS → Δalcohol model)**

**MCS → Δ alcohol model**	**Age and sex adjusted**		**Fully adjusted**^**b**^	
	**Alcohol**	**MCS**	**Alcohol**	**MCS**
Fixed effects				
Intercept	17.11 (16.69 to 17.53)	51.54*** (51.28 to 51.79)	17.58 (16.64 to18.52)	53.41*** (52.81 to 54.00)
Slope (α)	21.46** (8.50 to 34.43)	4.96 (-8.62 to 18.54)	23.31*** (11.00 to 35.62)	7.20 (-5.55 to 19.96)
Autoproportional (β)	-0.50*** (-0.61 to -0.40)	-0.07 (-0.33 to 0.19)	-0.50*** (-0.60 to -0.41)	-0.11 (-0.35 to 0.12)
Coupling (γ)	**–**	-0.30* (-0.53 to -0.06)	**–**	-0.31** (-0.52 to -0.10)
Random effects				
Residual variance	35.77*** (34.23 to 37.3)	35.02*** (33.51 to 36.54)	35.77*** (34.25 to 37.29)	34.94*** (33.45 to 36.42)
Intercept variance	177.95*** (170.31 to 185.58)	46.91*** (43.70 to 50.11)	144.21*** (137.72 to 150.71)	39.66*** (36.75 to 42.58)
Slope variance	26.26*** (12.55 to 39.98)	2.31*** (1.27 to 3.34)	23.66*** (12.24 to 35.08)	1.88** (0.81 to 2.95)
Intercept/slope correlation	0.69***	-0.30	0.67***	-0.12
Intercepts correlation	-0.02		0.02	
Slopes correlation	-0.11		-0.02	
Alcohol intercept, MCS slope correlation	-0.05		-0.06	

LCS, latent change score; MCS, mental health component score.

*** *P* < 0.001; ** *P* < 0.01; * *P* < 0.05.

^a^n = 6,330.

^b^Fully adjusted = age (centered around the sample mean), sex (male referent group), ethnicity (white (referent) versus non-white), socioeconomic status (defined by most recent recorded employment grade – entered as a linear term with high (referent), intermediate and low categories), marital status (married/cohabiting (referent) versus other), highest educational qualification (University (referent), post-secondary, secondary or no qualifications – entered as a continuous variable), economic activity (active (referent) versus inactive (merging retired and unemployed groups together)), social network (centered around the mean score), current smoking status (no (referent) versus yes), level of physical activity (active (referent), moderately active or low – entered as a linear term), CAGE caseness (no case (referent) versus case), use of anti-depressant medication was also controlled for (no (referent) versus current), self-reported long-standing physical illness (no (referent) versus yes), belonging to the lowest sex-specific SF-36 physical health component quartile (no (referent) versus yes), known diabetes (no (referent) versus yes), coronary heart disease (no (referent) versus yes), stroke (no (referent) versus yes), transient ischemic attack (no (referent) versus yes), total serum cholesterol (centered around the sample mean), systolic and diastolic blood pressure (centered around their mean values), a resting heart rate > 80 beats/minute (no (referent) versus yes) and body mass index (centered around the sample mean).

**Table 4:** **Fixed effect parameter estimates (95% confidence intervals) for other LCS model specifications of weekly alcohol consumption and mental health symptoms in the Whitehall II study**^**a**^

	**Age and sex adjusted**		**Fully adjusted**^**b**^	
	**Alcohol**	**MCS**	**Alcohol**	**MCS**
Baseline				
Intercept	17.15*** (16.73 to 17.57)	51.57*** (51.31 to 51.82)	17.63*** (16.69 to 18.57)	53.46*** (52.86 to 54.05)
Slope (α)	4.82*** (3.26 to 6.39)	5.34 (-8.16 to 18.85)	5.22*** (3.56 to 6.89)	7.77 (-4.69 to 20.24)
Autoproportional (β)	-0.43*** (-0.53 to -0.33)	-0.08 (-0.34 to 0.18)	-0.42*** (-0.52 to -0.33)	-0.12 (-0.36 to 0.11)
Coupling (γ)	–	–	–	–
Alcohol → ΔMCS model				
Intercept	17.15*** (16.73 to 17.57)	51.57*** (51.31 to 51.83)	17.63*** (16.7 to 18.57)	53.47*** (52.87 to 54.06)
Slope (α)	4.83*** (3.26 to 6.39)	7.86 (-8.21 to 23.94)	5.23*** (3.56 to 6.89)	10.82 (-3.90 to 25.55)
Autoproportional (β)	-0.43*** (-0.53 to -0.33)	-0.12 (-0.41 to 0.17)	-0.43*** (-0.52 to -0.33)	-0.17 (-0.42 to 0.09)
Coupling (γ)	-0.03 (-0.15 to 0.09)	–	-0.04 (-0.15 to 0.08)	–
Reciprocal Δ model				
Intercept	17.11*** (16.69 to 17.54)	51.55*** (51.29 to 51.81)	17.59*** (16.65 to 18.53)	53.42*** (52.83 to 54.02)
Slope (α)	21.49** (9.07 to 33.92)	8.72 (-6.03 to 23.47)	23.20*** (11.42 to 34.99)	11.05 (-2.70 to 24.79)
Autoproportional (β)	-0.50*** (-0.60 to -0.40)	-0.13 (-0.40 to 0.14)	-0.50*** (-0.59 to -0.40)	-0.17 (-0.41 to 0.08)
Coupling (γ)	-0.05 (-0.16 to 0.06)	-0.30** (-0.52 to -0.07)	-0.06 (-0.16 to 0.05)	-0.31** (-0.52 to -0.11)

LCS, latent change score; MCS, mental health component score.

*** *P* < 0.001; ** *P* < 0.01; * *P* < 0.05.

^a^n = 6,330.

^b^Fully adjusted = age (centered around the sample mean), sex (male referent group), ethnicity (white (referent) versus non-white), socioeconomic status (defined by most recent recorded employment grade – entered as a linear term with high (referent), intermediate and low categories), marital status (married/cohabiting (referent) versus other), highest educational qualification (University (referent), post-secondary, secondary or no qualifications – entered as a continuous variable), economic activity (active (referent) versus inactive (merging retired and unemployed groups together)), social network (centered around the mean score), current smoking status (no (referent) versus yes), level of physical activity (active (referent), moderately active or low – entered as a linear term), CAGE caseness (no case (referent) versus case), use of anti-depressant medication was also controlled for (no (referent) versus current), self-reported long-standing physical illness (no (referent) versus yes), belonging to the lowest sex-specific SF-36 physical health component quartile (no (referent) versus yes), known diabetes (no (referent) versus yes), coronary heart disease (no (referent) versus yes), stroke (no (referent) versus yes), transient ischemic attack (no (referent) versus yes), total serum cholesterol (centered around the sample mean), systolic and diastolic blood pressure (centered around their mean values), a resting heart rate > 80 beats per minute (no (referent) versus yes) and body mass index (centered around the sample mean).

As the association was robust to adjustment for confounding factors, only the fully adjusted estimates will be discussed here (but age and sex, as well as fully adjusted estimates are presented). Furthermore, only parameters of primary interest will be highlighted.

#### ***No coupling (baseline) model***

The top third of Table 4 refers to the baseline model (in which alcohol use and mental health do not influence changes in each other). A significant autoproportional effect for alcohol consumption was found (β = -0.42, CI -0.52 to -0.33) but not for mental health. The coefficient was negative, indicating that those drinking more made greater reductions in their alcohol consumption between phases.

#### ***Alcohol consumption producing change in mental health model***

The middle third of Table 4 shows estimates for a model where alcohol use affected upcoming change in mental health, but mental health had no effect on change in alcohol consumption. The alcohol autoproportional effect was significant (β = -0.43, CI -0.52 to -0.33) but not the mental health parameter. The coupling parameter was also non-significant. This model was compared with the baseline model, but offered no significant improvement in fit (Table 2).

#### ***Mental health producing change in alcohol consumption model***

The estimates concerning the model in which mental health scores affected upcoming change in alcohol consumption, but alcohol consumption had no effect on changes in mental health are presented in Table 3. A significant autoproportional effect was found for alcohol consumption (β = -0.50, CI -0.60 to -0.40) but not mental health. The coupling parameter was significant (γ = -0.31, CI -0.52 to -0.10) in this instance, and was negative, meaning that those with better mental health made greater reductions in their drinking. This model was an improvement over the baseline model (Table 2; *P* < 0.01).

#### ***Dynamic/reciprocal change model***

The final third of Table 4 shows estimates from a model in which both alcohol consumption and mental health scores are able to affect change in the alternative variable. As in previous models, a significant autoproportional effect was found for alcohol consumption (β = -0.50, CI -0.59 to -0.40) but not mental health. The coupling parameter from previous phase mental health to change in alcohol consumption remained significant (γ = -0.31, CI -0.52 to -0.11), whereas the effect of previous occasion alcohol consumption was not associated with changes in mental health. This model offered little improvement in fit over the previous model (Table 2), indicating that the model in which mental health influences changes in alcohol consumption but not *vice versa* is the best fit to the data.

It is necessary to jointly interpret the estimates in Table 3 to fully appreciate the dynamics of the alcohol use and mental health system because parameters are dependent on each other [54,55,57,62]. Concentrating on the fully adjusted estimates, to predict change, Equation 4 in Additional file 1 would be adapted to remove the coupling parameter from previous phase alcohol consumption to changes in mental health, resulting in a final change equation (conditional on other covariates in the model; for coefficients, see Additional file 3: Table S3A) of:

$$\begin{array}{ll} {\Delta \text{Alcohol}}_{\mathrm{it}} & =23.31\pm 4.86-0.50\times {\text{Alcohol}}_{\mathrm{it}-1}\\ & -0.31\times {\text{MCS}}_{\mathrm{it}-1}\\ {\Delta \text{MCS}}_{\mathrm{it}} & =7.20\pm 1.37-0.11\times {\text{MCS}}_{\mathrm{it}-1} \end{array}$$

**Figure 2:**
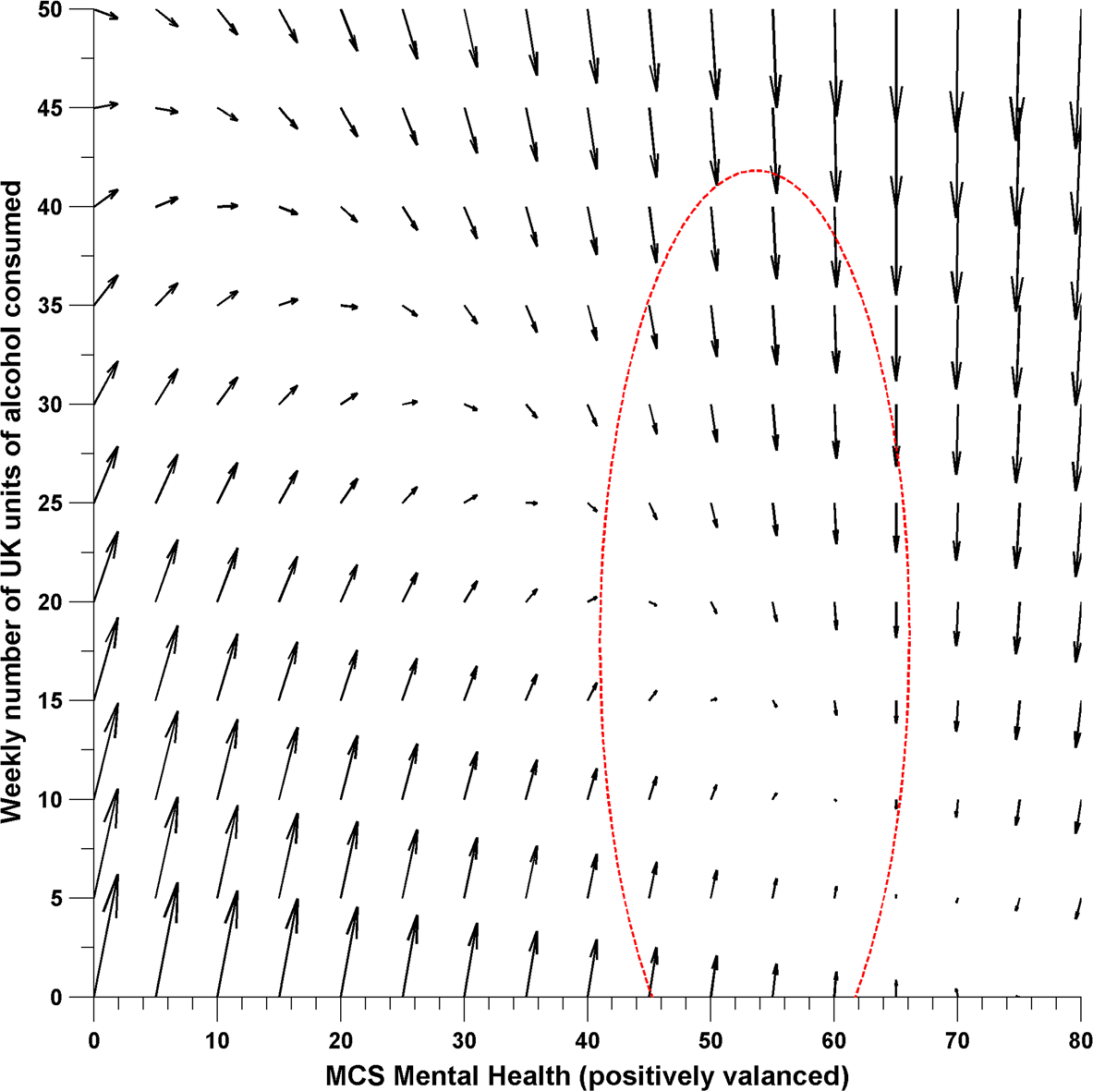
**Vector field showing joint movements between weekly alcohol consumption and mental health scores in Whitehall II as a function of the mental health producing change in weekly alcohol consumption system.** Ellipsoid reflects 95% of the data.

The expected change in both mental health scores and UK units of alcohol consumed between phases can then be plotted within a vector field [63] (Figure 2). This figure displays the direction and magnitude of change in both variables for a given set of starting co-ordinates. The ellipsoid reflects where 95% of the data lay.

## Discussion

### Summary and interpretation of findings

A series of LCS models were estimated to test lag-leading and reciprocal relationships between weekly number of UK units consumed and mental health. In both minimally adjusted and fully adjusted models, it was found that a model in which mental health was specified as the leading indicator of change gave the best fit (Table 2, Table 3).Plotting the parameters of this model in a vector field (Figure 2) demonstrates the complex relationship between weekly alcohol consumption and mental health, and also helps to visualize the correlation between the mental health intercept and alcohol slope, which is difficult to interpret in isolation. It shows that participants who initially had poor mental health and low alcohol consumption increased their consumption between phases, whereas those with good to adequate mental health who drank at higher levels tended to decrease their consumption between phases while their mental health scores remained relatively stable. Furthermore, it shows that participants with poor mental health and high alcohol consumption on the previous occasion had a shallower decline in their consumption than those with good mental health drinking the same amount. This indicates that in addition to fuelling increases in alcohol consumption among low-level consumers, poor mental health may also be a maintaining factor for heavy alcohol consumption.

### Comparison with other work

Our findings contradict the most recently published review on the relationship between alcohol use and depression [10], which concluded that increasing involvement with alcohol raises the risk of depression by two-fold. This review was, however, met with criticism [64,65] for primarily being based on previous work by the authors themselves [9]. Furthermore, the review focused on alcohol-use disorders and major depression. Our work is therefore not directly comparable. As outlined earlier, we chose to focus on sub-syndrome symptoms of mental health and alcohol consumption (not problem consumption) as there has been a distinct lack of work exploring actual alcohol consumption (that is, what people drink) in this relationship; previous interest has largely been on the relationship between alcohol-use disorders and major depression. This makes drawing comparisons between our work and others complicated. It may be that there is something about the symptoms of problematic alcohol consumption that increases the risk of having [6,7,66-71] or developing [9,10,72,73] depression, independent of the amount of alcohol consumed [46,74]. Recent work has shown that individuals who self-medicate symptoms of anxiety [75] or depression [12] with alcohol have an increased risk of developing (persistent) alcohol dependence. Therefore, it could be that the relationship we observed is part of a larger complex system involving a transition from sub-syndromal symptoms of mental health influencing changes in alcohol consumption (as in our analyses) until a certain threshold is reached, at which symptoms of alcohol dependence take over and increase the risk of developing clinical disorders [9,10]; that is, there are two separate dynamic systems at play that influence alcohol consumption and mental health pre and post clinical disorder.

### Strengths and weaknesses

The approach that we took to modeling the relationship between alcohol use and mental health longitudinally utilized multiple measurement occasions to model change in both variables over time, which is known to improve the accuracy of estimated change [58,76]. Previous work has also shown that it is important to consider variability in alcohol consumption [77], and the LCS model methodology directly incorporated individual change both in the total weekly alcohol consumption and in mental health. Furthermore, the method we used allowed for the effect of alcohol consumption on mental health and *vice versa* to be estimated simultaneously in the same model.

There are, however, several limitations of our study. First, data from phase 5 were used as the starting point, and it is possible that selective attrition may have occurred between the 'true' baseline (phase 1) and the baseline used in these analyses. This would result in a healthier cohort of participants being used to estimate the final model parameters, reducing the generalizability of the findings [78,79]. Similarly, we used data from the Whitehall II cohort of British civil servants, which is not a representative sample of the general population. Work published using Whitehall II data has been highly influential in epidemiology and public health, shaping research agendas on social inequalities in health [80] and improving the understanding of the etiology of disease [81] but this limitation should be noted when considering the generalizability of our findings.

Second, one of the major concerns in alcohol epidemiology is measurement error in self-reported alcohol consumption [82]. It is acknowledged that self-reported measures of consumption are likely to be biased [82-88], and therefore effect estimates obtained may actually be underestimates of the true association of interest. The use of latent variables (upon which LCS models are based; see Additional file 1) has been advocated in the field of alcohol epidemiology [89] to account for this known measurement error. Additionally, the MCS scale of the SF-36 is not solely concerned with psychiatric symptoms but also with mental health-related quality of life (although evidence exists to suggest that high MCS scores are associated with clinical depression [40,90,91]). It is possible that the relationship between alcohol intake and mental health might differ if other psychiatric questionnaires were used to define symptoms of mental health, or if the distinction was made between symptoms of depression and anxiety. However, it is argued that in practice it is difficult to effectively determine specific characteristics of depression from symptoms of, for example, anxiety using self-report measures of symptoms because of the considerable heterogeneity of symptoms between disorders (that is, self-reported symptoms often reflect a comorbidity between depression and other mood/stress-related disorders [92-95]). This has led some investigators to conclude that self-report measures of mental health symptoms at a population level merely reflect a single underlying latent construct of psychological distress [96-99].

Another issue concerning the main measures used in this study is that they refer to different time periods; information on alcohol consumption pertained to the previous week whereas information on mental health symptoms referred to the previous 4 weeks. It is possible that this discrepancy in the period of reference may have biased our findings. For example, smaller studies looking at the relationship between mood and alcohol on a daily basis have shown that increased alcohol consumption is associated with decreased happiness on the following day, and that symptoms of sadness are associated with decreased consumption on the next day [100]. These findings contrast with our own, and highlight the importance of the timeframe used in determining the best-fitting temporal sequence between alcohol consumption and mental health.

Furthermore, the competing models we specified allowed only for the previous occasion's alcohol and/or mental health score to influence change in the alternative variable by the next occasion. It is plausible that the relationship might have differed if we had allowed for longer lag specifications, as it may be that the relationship between alcohol intake and mental health takes longer to manifest (that is, the current specification of a single cross-lagged effect may fit the relationship between mental health influencing alcohol intake better than the relationship between alcohol consumption influencing mental health symptoms).

Additionally, there was greater variation in the measure of weekly alcohol intake than in that of mental health. It could be argued that this could also be a possible explanation as to why alcohol consumption was not found to be significantly related to changes in mental health. It may be that within a dynamic system that it is more difficult to effectively predict changes in one variable using a highly erratic alternative exposure. It is conceivable that the reciprocal model might have been of best fit had both measures been relatively stable over time.

A further methodological limitation is that we controlled only for baseline covariate values, and it is possible that their values changed over time. For example, comorbidities may have developed after the first measurement occasion. Health behaviors such as physical activity and smoking could also vary over time, and the changing status of these variables could all be confounders of the subsequent effects of alcohol on mental health and *vice versa*. However, factoring in changes in the covariate structure over time could be problematic within the current framework, because changes in some values, for example, systolic blood pressure, could be a direct consequence of previous alcohol consumption or mental health status, and thus be considered as intermediate confounders [101].

### Implications and directions for future work

We identified that the dominant process underlying the dynamic relationship between alcohol consumption and mental health at a population level is mental health. Consequently, it could be inferred that targeting interventions to those with poor mental health (as well as introducing measures to ensure that those with normal/good mental health do not deteriorate) would have a beneficial effect in terms of reducing heavy drinking. This may also elicit favorable knock-on effects in terms of improving general physical health and reducing the risk of chronic diseases, as heavy drinking itself is associated with an increased risk of a range of health problems [102,103] including cardiovascular disease [104-109], cancer, [110,111] and mortality [47,112-114]. Furthermore, the finding that mental health affects alcohol consumption may shed some light on the growing literature examining common mental disorders as risk factors for cardiovascular disease [115-119] and all-cause mortality [120-122], because alcohol consumption may be one of many mediators in this relationship.

This work provides further support that on-going efforts to improve mental health at a population level are vital to public health [123,124]. The proposed implementation strategy [124] seeks both to tackle he social determinants of mental health [125] and to target individuals who are at high risk. To do so, a number of avenues will be pursued, including tackling inequalities in access to services (and ensuring equality in the level of service provided). In addition, conscious efforts are being made to tackle the stigma surrounding mental health issues; perhaps if individuals feel more comfortable talking about their mental health problems or seeking treatment for them, then they will not turn to alcohol as a form of self-medication.

Others may, however, be more cynical of our findings and take them to indicate that 1) consuming large amounts of alcohol is acceptable as it does not increase the risk of developing mental health problems, and 2) that it is reasonable to self-medicate with alcohol in response to psychological distress, as it will not worsen symptoms. However, it would be unwise to use our findings as a justification for drinking in a hazardous manner. Although a person’s mental health may not worsen, as highlighted above, increased alcohol consumption would heighten their risk of developing other disorders.

Regarding future work, it is important to examine the role of drinking pattern as well as to provide closer scrutiny of age (e.g. adolescent and elderly populations), sex, socioeconomic, and cultural differences in the dynamic relationship between alcohol consumption and mental health. It is also important that subsequent studies should examine the extent to which time-varying/modified confounding may explain the association observed using appropriate analytic methods [101]. Furthermore, it is also imperative that potential physiological and psychosocial mechanisms, both occurring alongside and precipitating (immediately or earlier in life) the parallel development of both trajectories are studied. This has been acknowledged by others in the field [10,64,65]. Understanding the factors that trigger increased alcohol consumption in the presence of poor mental health will allow for more effective interventions to be developed, both in terms of treatment and primary prevention.

## Conclusions

Mental health appears to be the leading indicator of change in the dynamic longitudinal relationship between mental health and weekly alcohol consumption in this middle-aged, mostly white, male, and well-educated sample of individuals. In addition to increasing alcohol intake among low-level consumers, poor mental health may also be a maintaining factor for sustained high alcohol intake in heavy alcohol consumers. Our findings therefore indicate that on-going efforts to improve mental health at a population level may also help to reduce hazardous alcohol consumption. Future work should seek to examine whether there are critical levels of alcohol consumption at which different dynamic relationships operate between alcohol-related behavior and mental health, specifically focusing on heterogeneities in the dynamic processes between alcohol intake and symptoms of alcohol dependence, and mental health pre and post clinical disorder to try and better capture the possible discontinuous progression from sub-syndrome behavior to clinically relevant outcomes.

## Competing interests

None of the authors have any competing interests.

## Author contributions

SB and AB devised the research question. SB analysed the data and completed the first draft of the manuscript. AB provided important additional comments on the initial manuscript. Both SB and AB agreed on the decision to submit the final manuscript. SB had full access to all of the data in the study, and takes responsibility for the integrity of the data and the accuracy of the data analysis. All authors read and approved the final manuscript.

## Pre-publication history

The pre-publication history for this paper can be accessed here:

http://www.biomedcentral.com/1741-7015/12/91/prepub

## Supplementary Material

Additional file 1 **Description of latent change score methodology. Figure S1A.** A path diagram representing a bivariate latent change score model. **Table S1A** – Definition of parameters depicted in Additional file 1: Figure S1A. Click here for file

Additional file 2 **Table S2A.** – Detailed parameter estimates (95% confidence intervals) for all latent change score (LCS) model specifications of weekly alcohol consumption and mental health symptoms in the Whitehall II study. Click here for file

Additional file 3 **Description of covariate coefficients (95% CI) for fully-adjusted mental health component score (MCS) producing change in alcohol consumption model. Table S3A** - Covariate coefficients (95% CI) for fully-adjusted MCS producing change in alcohol consumption model. Click here for file
