# Supplementary material for: An exploration of the dynamic longitudinal relationship between mental health and alcohol consumption: a prospective cohort study
Source: BMC Med. 2014 Jun 3;12:91. doi: 10.1186/1741-7015-12-91 (PMC4053287; doi:10.1186/1741-7015-12-91)
Supplement: Additional file 1 — Description of latent change score methodology. Figure S1A. A path diagram representing a bivariate latent change score model. Table S1A – Definition of parameters depicted in Additional file 1: Figure S1A. [file 1741-7015-12-91-S1.pdf]

Supplementary Data 1 (Bell & Britton)

Online-only table of contents

Supplementary data 1

- **Description of latent change score methodology**
- **Supplementary Figure 1A - A path diagram representing a bivariate latent change score model**
- **Supplementary Table 1A - Definition of parameters depicted in Supplementary Figure 1A**

Supplementary data 1

Description of latent change score methodology

Here we will describe LCS models based on two processes with observed variables *Y* and *X* at time *t* for individual *i* (represented in graphical form in Supplementary Figure 1A with parameters defined in Supplementary Table 1A).

To begin, it is important to detail how latent difference scores are defined. Under classical true score theory it is assumed that each observed raw score (*Y*) can be disintegrated into a true underlying latent score *y* or *x* plus a source of unrelated error *e*. Therefore, one can express the observed score at any time point *t*, as:

$$Y_{it} = y_{it} + e_{yit}$$
$$X_{it} = x_{it} + e_{xit}$$

Equation 1

These true scores can then be used to define the present state of each variable as a function of its preceding state plus changes, using:

$$y_{it} = y_{it-1} + \Delta y_{it}$$
$$x_{it} = x_{it-1} + \Delta x_{it}$$

Equation 2

From this equation, the development of observed variables *Y* and *X* for a person *i* at a specified time *t* can be expressed as a function of an originally observed score (*y*0 and *x*0)

plus the linear accumulation of latent changes (Δy and Δx) until that point in time in addition to residual error (e_y and e_x), using:

$$Y_{it} = y_{i0} + \left(\sum_{k=1}^{t} \Delta_{yki} \right) + e_{yit}$$
$$X_{it} = x_{i0} + \left(\sum_{k=1}^{t} \Delta_{xki} \right) + e_{xit}$$

Equation 3

Following from this a model for the latent change scores can be written as a product of multiple components. One common specification is:

$$\Delta y_{it} = \alpha_y \times y_{is} + \beta_y \times y_{it-1} + \gamma_y \times x_{it-1}$$
$$\Delta x_{it} = \alpha_x \times x_{is} + \beta_x \times x_{it-1} + \gamma_x \times y_{it-1}$$

Equation 4

Whereby change in a variable (Δ) is a function of three main components: a constant amount (α) which is associated with the additive scores/slopes of y_{is} and x_{is} (the sum of latent changes over time), a quantity proportional to the previous state of itself (β) – in many ways representing a self-feedback loop, and an amount proportional to the previous state of the alternative variable (γ). Placing certain constraints on parts of this model allow for specific hypotheses to be tested.

For example, constraining the coupling parameter (γ) from *x* to *y* to be zero, while estimating the parameter from *y* to *x*, would model a leading effect of *y* to changes in *x*. Alternatively, one is able to use Equation 4 above (both coupling parameters freed) to explore whether there

is a reciprocal dynamic relationship over time between both variables. Note that the dynamics of the system are brought about by jointly estimating and interpreting these equations together as the model parameters are dependent on each other [1–4].

It is also important to note that while LCS models are usually specified as linear models, nonlinear trajectories can be accommodated through the use of first differences because at each time point the proportional and coupling parameters are multiplied by scores from the previous measurement occasion which alter over time. The result is that even in a model where the coefficients are assumed to be static over time the actual effects are compounded across occasions as a result of being multiplied by shifting values.

References

1. McArdle JJ, Grimm KJ (2010) Five Steps in Latent Curve and Latent Change Score Modeling with Longitudinal Data. Longitudinal Research with Latent Variables. Heidelberg, Berlin: Springer. pp. 245–273.

2. McArdle J (2011) Longitudinal dynamic analyses of cognition in the health and retirement study panel. AStA Advances in Statistical Analysis 95: 453–480. doi:10.1007/s10182-011-0168-z.

3. McArdle JJ, Hamagami F (2001) Latent Difference Score Structural Models for Linear Dynamic Analyses With Incomplete Longitudinal Data. New Methods for the Analysis of Change. Washington, DC: American Psychological Association. pp. 137–176.

4. Hamagami F, McArdle JJ (2001) Advanced Studies of Individual Differences Linear Dynamic Models for Longitudinal Data Analysis. New Developments and Techniques in

Structural Equation Modeling. Mahwah, New Jersey: Lawrence Erlbaum Associates, Inc. pp. 203–246.

5. Kline RB (2010) Principles and Practice of Structural Equation Modeling. 3rd ed. New York, USA: The Guildford Press. 432 p.

6. Ferrer E, McArdle JJ (2010) Longitudinal Modeling of Developmental Changes in Psychological Research. Current Directions in Psychological Science 19: 149 –154. doi:10.1177/0963721410370300.

Supplementary Figure 1A. A path diagram representing a bivariate latent change score model[1]

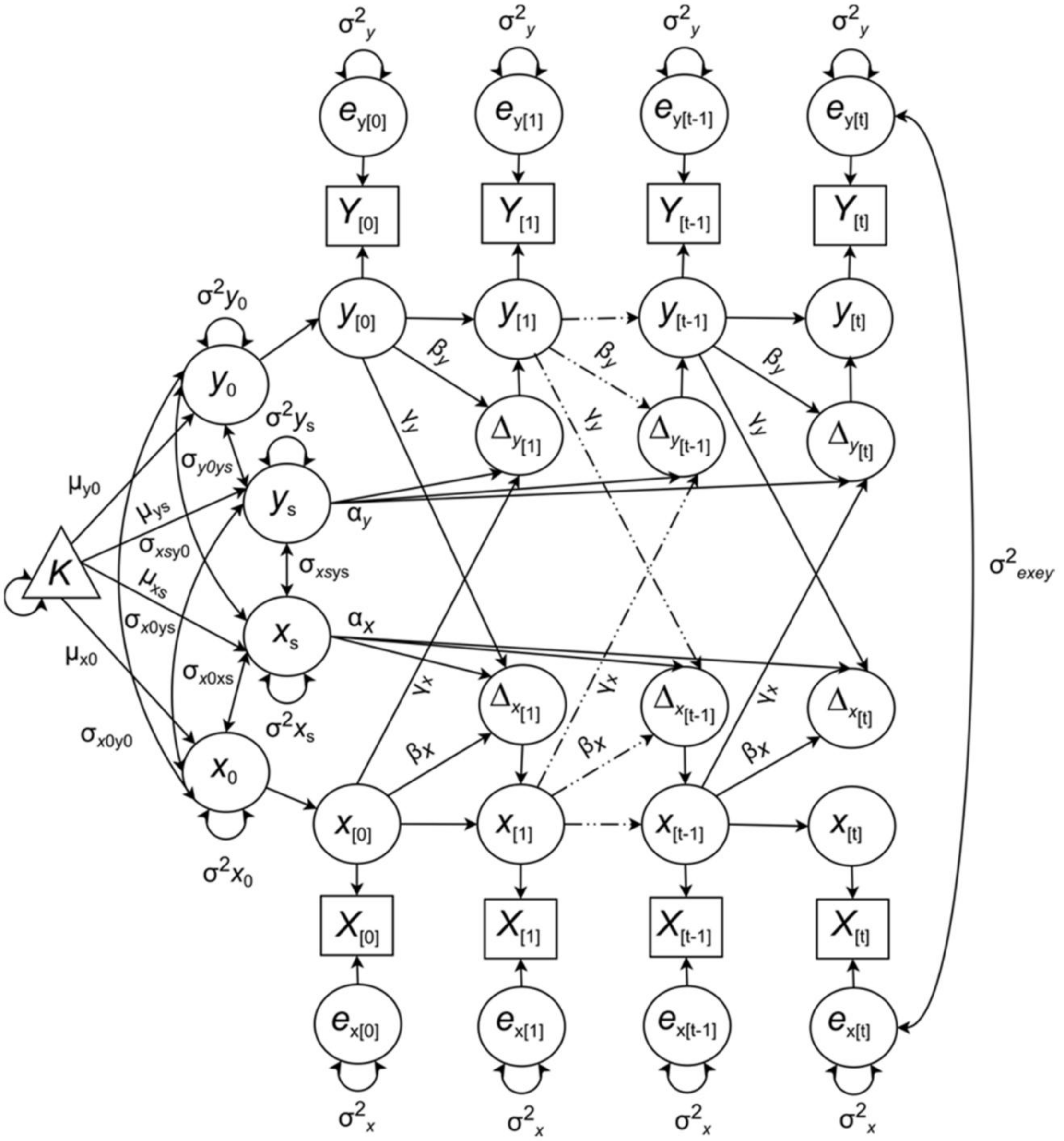

---

[1] Using standard structural equation modelling graphical notation [5]: squares represent observed variables and circles represent latent variables. Single-headed arrows indicate regression coefficients or intercepts. Double-headed arrows represent variance or covariance terms. The triangle represents a constant (set to 1 for identification purposes).

Supplementary Table 1A – Definition of parameters depicted in Supplementary Figure 1A [2]

Parameter	Definition
Y_{it}, X_{it}	Observed values for variables Y and X
y_{it}, x_{it}	Latent scores for variables Y and X
Δy_{it}, Δx_{it}	Latent changes in (latent) y and x
$y0_i$, $x0_i$	Latent intercept for variables Y and X
ys_i, xs_i	Latent slope (additive scores) for variables Y and X
αy, αx	Additive component of change for variables y and x
βy, βx	Autoproportional change parameter for variables y and x
γy, γx	Coupling parameter for variables y and x
μy0, μx0	Mean of initial conditions for variables Y and X
μys, μxs	Mean of additive scores for variables Y and X
$\sigma^2 y0$, $\sigma^2 x0$	Variance of initial conditions of Y and X
$\sigma^2 ys$, $\sigma^2 xs$	Variance of additive scores of Y and X
$\sigma^2 ey$, $\sigma^2 ex$	Residual variance of initial conditions of Y and X
σy0, x0	Covariance of initial conditions of Y and X
σys, xs	Covariance of additive scores of Y and X
σey, ex	Covariance of residuals from Y and X
K	Constant to estimate means and intercepts (set to 1)

[2] Presentation of Supplementary Figure 1A alongside Supplementary Table 1A adapted from [6]
