# Supplementary material for: An exploration of the dynamic longitudinal relationship between mental health and alcohol consumption: a prospective cohort study
Source: BMC Med. 2014 Jun 3;12:91. doi: 10.1186/1741-7015-12-91 (PMC4053287; doi:10.1186/1741-7015-12-91)
Supplement: Additional file 2 — Table S2A. – Detailed parameter estimates (95% confidence intervals) for all latent change score (LCS) model specifications of weekly alcohol consumption and mental health symptoms in the Whitehall II study. [file 1741-7015-12-91-S2.pdf]

Supplementary Data 2 (Bell & Britton)

Online-only table of contents

Supplementary data 2

- **Supplementary Table 2A – Detailed parameter estimates (95% confidence intervals) for all LCS model specifications of weekly alcohol consumption and mental health symptoms in the Whitehall II study**

Supplementary Table 2A - Detailed parameter estimates (95% confidence intervals) for all LCS model specifications of weekly alcohol consumption and mental health symptoms in the Whitehall II study

**Baseline**	**Age and sex adjusted**		**Fully adjusted**	
Fixed effects				
	**Alcohol**	**MCS**	**Alcohol**	**MCS**
Intercept	17.15*** (16.73, 17.57)	51.57*** (51.31, 51.82)	17.63*** (16.69, 18.57)	53.46*** (52.86, 54.05)
Slope (α)	4.82*** (3.26, 6.39)	5.34 (-8.16, 18.85)	5.22*** (3.56, 6.89)	7.77 (-4.69, 20.24)
Autoproportional (β)	-0.43*** (-0.53, -0.33)	-0.08 (-0.34, 0.18)	-0.42*** (-0.52, -0.33)	-0.12 (-0.36, 0.11)
Coupling (γ)				
Random effects				
Residual variance	36.29*** (34.78, 37.80)	34.89*** (33.38, 36.39)	36.34*** (34.82, 37.85)	34.77*** (33.3, 36.24)
Intercept variance	176.69*** (169.11, 184.27)	47.38*** (44.21, 50.55)	143.00*** (136.55, 149.45)	40.15*** (37.26, 43.04)
Slope variance	15.93*** (8.23, 23.63)	2.41*** (1.4, 3.43)	13.88*** (7.51, 20.25)	2.02*** (0.90, 3.14)
Intercept/slope correlation	0.69***	-0.29	0.65***	-0.10
Intercepts correlation	-0.03		0.01	
Slopes correlation	-0.13*		-0.12	
Alcohol intercept, MCS slope correlation	-0.04		-0.04	
MCS intercept, alcohol slope correlation	0.03		0.05	
**Alcohol → Δ MCS model**	**Age and sex adjusted**		**Fully adjusted**	
Fixed effects				
	**Alcohol**	**MCS**	**Alcohol**	**MCS**
Intercept	17.15*** (16.73, 17.57)	51.57*** (51.31, 51.83)	17.63*** (16.70, 18.57)	53.47*** (52.87, 54.06)
Slope (α)	4.83*** (3.26, 6.39)	7.86 (-8.21, 23.94)	5.23*** (3.56, 6.89)	10.82 (-3.90, 25.55)
Autoproportional (β)	-0.43*** (-0.53, -0.33)	-0.12 (-0.41, 0.17)	-0.43*** (-0.52, -0.33)	-0.17 (-0.42, 0.09)
Coupling (γ)	-0.03 (-0.15, 0.09)		-0.04 (-0.15, 0.08)	

Supplementary Table 2A. Parameter estimates (95% confidence intervals) for LCS models of weekly alcohol consumption and mental health symptoms in the Whitehall II study (continued)				
Random effects				
Residual variance	36.30*** (34.78, 37.81)	34.79*** (33.23, 36.34)	36.35*** (34.83, 37.86)	34.65*** (33.14, 36.16)
Intercept variance	176.68*** (169.10, 184.27)	47.55*** (44.30, 50.79)	142.98*** (136.53, 149.44)	40.34*** (37.39, 43.29)
Slope variance	15.93*** (8.22, 23.64)	2.50** (0.98, 4.03)	13.89*** (7.51, 20.27)	2.32* (0.09, 4.56)
Intercept/slope correlation	0.69***	-0.14	0.65***	0.08
Intercepts correlation	-0.02		0.02	
Slopes correlation	0.06		0.11	
Alcohol intercept, MCS slope correlation	0.19		0.23	
MCS intercept, alcohol slope correlation	0.03		0.05	
**MCS → Δ Alcohol model**	**Age and sex adjusted**		**Fully adjusted**	
Fixed effects				
	**Alcohol**	**MCS**	**Alcohol**	**MCS**
Intercept	17.11 (16.69, 17.53)	51.54*** (51.28, 51.79)	17.58 (16.64, 18.52)	53.41*** (52.81, 54)
Slope (α)	21.46** (8.50, 34.43)	4.96 (-8.62, 18.54)	23.31*** (11.00, 35.62)	7.20 (-5.55, 19.96)
Autoproportional (β)	-0.50*** (-0.61, -0.40)	-0.07 (-0.33, 0.19)	-0.50*** (-0.60, -0.41)	-0.11 (-0.35, 0.12)
Coupling (γ)		-0.30* (-0.53, -0.06)		-0.31** (-0.52, -0.10)
Random effects				
Residual variance	35.77*** (34.23, 37.30)	35.02*** (33.51, 36.54)	35.77*** (34.25, 37.29)	34.94*** (33.45, 36.42)
Intercept variance	177.95*** (170.31, 185.58)	46.91*** (43.70, 50.11)	144.21*** (137.72, 150.71)	39.66*** (36.75, 42.58)
Slope variance	26.26*** (12.55, 39.98)	2.31*** (1.27, 3.34)	23.66*** (12.24, 35.08)	1.88** (0.81, 2.95)
Intercept/slope correlation	0.69***	-0.30	0.67***	-0.12
Intercepts correlation	-0.02		0.02	
Slopes correlation	-0.11		-0.02	
Alcohol intercept, MCS slope correlation	-0.05		-0.06	

Supplementary Table 2A. Parameter estimates (95% confidence intervals) for LCS models of weekly alcohol consumption and mental health symptoms in the Whitehall II study (continued)				
MCS intercept, alcohol slope correlation	0.37**		0.40***	
**Reciprocal Δ model**	**Age and sex adjusted**		**Fully adjusted**	
Fixed effects				
	**Alcohol**	**MCS**	**Alcohol**	**MCS**
Intercept	17.11*** (16.69, 17.54)	51.55*** (51.29, 51.81)	17.59*** (16.65, 18.53)	53.42*** (52.83, 54.02)
Slope (α)	21.49** (9.07, 33.92)	8.72 (-6.03, 23.47)	23.2*** (11.42, 34.99)	11.05 (-2.7, 24.79)
Autoproportional (β)	-0.50*** (-0.6, -0.4)	-0.13 (-0.4, 0.14)	-0.50*** (-0.59, -0.40)	-0.17 (-0.41, 0.08)
Coupling (γ)	-0.05 (-0.16, 0.06)	-0.30** (-0.52, -0.07)	-0.06 (-0.16, 0.05)	-0.31** (-0.52, -0.11)
Random effects				
Residual variance	35.77*** (34.23, 37.3)	34.86*** (33.32, 36.4)	35.78*** (34.25, 37.3)	34.77*** (33.26, 36.28)
Intercept variance	177.97*** (170.33, 185.6)	47.16*** (43.91, 50.42)	144.21*** (137.71, 150.71)	39.91*** (36.95, 42.87)
Slope variance	25.75*** (12.76, 38.73)	2.67* (0.51, 4.82)	23.05*** (12.28, 33.82)	2.42 (-0.04, 4.88)
Intercept/slope correlation	0.69***	-0.08	0.66***	0.09
Intercepts correlation	-0.02		0.03	
Slopes correlation	0.28		0.37	
Alcohol intercept, MCS slope correlation	0.32		0.33	
MCS intercept, alcohol slope correlation	0.38**		0.41***	

*** $p < 0.001$; ** $p < 0.01$; * $p < 0.05$
N=6,330

Fully adjusted = age (centred around the sample mean), sex (male referent group), ethnicity (white [referent] vs non-white), socioeconomic status (defined by last recorded employment grade – entered as a linear term with high [referent], intermediate and low categories), marital status (married/cohabiting [referent] vs other), highest educational qualification (University [referent], post-secondary, secondary or no qualifications – entered as a continuous variable), economic activity (active [referent] vs in-active

[merging retired and unemployed groups together]), social network (centred around the mean score), current smoking status (no [referent] vs yes), level of physical activity (active [referent], moderately active or low – entered as a linear term), CAGE caseness (no case [referent] vs case), use of anti-depressant medication was also controlled for (no [referent] vs current), self-reported long-standing physical illness (no [referent] vs yes), belonging to the lowest sex-specific SF-36 physical health component quartile (no [referent] vs yes), known diabetes (no [referent] vs yes), CHD (no [referent] vs yes), stroke (no [referent] vs yes), TIA (no [referent] vs yes), total serum cholesterol (centred around the sample mean), systolic and diastolic blood pressure (centred around their mean values), a resting heart rate > 80 beats per minute (no [referent] vs yes) and BMI (centred around the sample mean).
