# Supplementary material for: An exploration of the dynamic longitudinal relationship between mental health and alcohol consumption: a prospective cohort study
Source: BMC Med. 2014 Jun 3;12:91. doi: 10.1186/1741-7015-12-91 (PMC4053287; doi:10.1186/1741-7015-12-91)
Supplement: Additional file 3 — Description of covariate coefficients (95% CI) for fully-adjusted mental health component score (MCS) producing change in alcohol consumption model. Table S3A - Covariate coefficients (95% CI) for fully-adjusted MCS producing change in alcohol consumption model. [file 1741-7015-12-91-S3.pdf]

Supplementary Data 3 (Bell & Britton)

Online-only table of contents

Supplementary data 3

- **Description of covariate coefficients (95% CI) for fully-adjusted MCS → Δ in alcohol consumption model**
- **Supplementary Table 3A - Covariate coefficients (95% CI) for fully-adjusted MCS → Δ in alcohol consumption model**

Supplementary data 3

Description of covariate coefficients (95% CI) for fully-adjusted MCS → Δ in alcohol consumption model

Supplementary Table 3A shows the covariate coefficients for the model which was identified as being of best fit in Table 2 (of the main manuscript) - where mental health was specified as the leading indicator of change (estimates presented in Table 3 of the main manuscript). A number of covariates were related to weekly alcohol consumption and/or mental health intercepts and slopes.

To begin with covariates associated with participants intercepts, conditional on other covariates included in the model, age was negatively associated with the alcohol intercept (δ=-0.10, CI -0.18, -0.03) and positively associated with the mental health intercept (δ=0.30, CI 0.26, 0.35) meaning that older participants mean consumed less alcohol and had better mental health scores at baseline. Women were also more likely to have lower weekly alcohol consumption at baseline (δ=-7.43, CI -8.27, -6.59) and slightly poorer mental health too (δ=-0.80, CI -1.33, -0.28). Those from non-white ethnic groups consumed less alcohol at baseline (δ=-3.07, CI -4.56, -1.57). Socioeconomic status was negatively associated with both baseline alcohol consumption (δ=-2.04, CI -2.68, -1.39) and mental health scores (δ=-0.74, CI -1.15, -0.34) meaning that for each category lower than "high SES" participants consumed less alcohol and had poorer mental health scores. Educational attainment was associated with the mental health intercept (δ=0.30, CI 0.04, 0.56) with participants who had less than degree level education having better mental health. Those not in employment had lower levels of alcohol consumption at baseline (δ=-0.97, CI -1.84, -0.10). Social network was positively associated with both alcohol consumption (δ=0.19, CI 0.07, 0.31) and mental health scores

(δ=0.38, CI 0.30, 0.45) meaning that those with higher social network scores consumed more alcohol and had better mental health scores at baseline.

Smokers consumed more alcohol at baseline (δ=4.46, CI 3.34, 5.58) while those who were less physically active had lower mental health scores (δ=-0.62, -0.88, -0.37). Aspects of physical health associated with alcohol consumption at baseline included BMI (δ=0.20, CI 0.10, 0.31), total serum cholesterol (δ=1.58, CI 1.24, 1.92), systolic (δ=0.08, CI 0.05, 0.11) and diastolic (δ=-0.08, CI -0.13, -0.04) blood pressure – those with higher values were likely to consume greater amounts of alcohol (with the exception of diastolic blood pressure which was negatively associated with alcohol consumption). Physical health characteristics associated with lower (poorer) mental health intercepts included a history of longstanding illness (δ=-1.93, CI -2.37, -1.48) and antidepressant medication use (δ=-8.07, CI -9.37, -6.77) and while higher systolic blood pressure values were associated with higher mental health scores (δ=0.03, CI 0.01, 0.05). Problematic alcohol users consumed significantly more alcohol (δ=14.87, CI 13.79, 15.95) and had poorer mental health at baseline (δ=-3.14, CI -3.81, -2.46).

Moving onto covariates associated with alcohol and mental health slopes; being a woman (δ=-2.48, CI -3.33, -1.64), of non-white ethnicity (δ=-1.70, CI -2.56, -0.85) and of lower socioeconomic status (δ=-0.99, CI -1.43, -0.55) were all negatively associated with the alcohol slope. This means that they made less total change in their alcohol consumption over time. Not being in employment was negatively associated with the mental health slope (δ=-0.44, CI -0.74, -0.15). Participants social networks were positively associated with the alcohol slope (δ=0.13, CI 0.03, 0.23) as was smoking (δ=1.05, CI 0.37, 1.73) at baseline, while a history of longstanding illness was negatively associated with the alcohol slope (δ=-0.78, CI -1.30, -0.25) as was antidepressant medication use (δ=-2.32, -4.18 -0.46) and physical activity level (δ=-0.36, -0.60, -0.12). Belonging to the lowest sex-specific quartile

of physical functioning at baseline was negatively associated with the mental health slope (δ=-1.22, CI -1.52, -0.92) while BMI was positively associated with the mental health slope (δ=0.07, 0.03, 0.11). Total serum cholesterol (δ=0.34, 0.13, 0.55) and systolic blood pressure (δ=0.03, CI 0.01, 0.05) were positively associated with the alcohol slope. CAGE caseness (δ=3.24, CI 1.93, 4.54) at baseline was positively associated with the alcohol slope.

Supplementary Table 3A - Covariate coefficients (95% CI) for fully-adjusted MCS → Δ in alcohol consumption model

	Alcohol intercept	Alcohol slope	Mental health intercept	Mental health slope
**Age**	-0.10 (-0.18, -0.03)	~0.00 (-0.07, 0.06)	0.30 (0.26, 0.35)	-0.04 (-0.11, 0.03)
**Women**	-7.43 (-8.27, -6.59)	-2.48 (-3.33, -1.64)	-0.80 (-1.33, -0.28)	0.34 (0.03, 0.66)
**Non-white**	-3.07 (-4.56, -1.57)	-1.70 (-2.56, -0.85)	-0.63 (-1.56, 0.31)	-0.21 (-0.75, 0.32)
**SES**	-2.04 (-2.68, -1.39)	-0.99 (-1.43, -0.55)	-0.74 (-1.15, -0.34)	-0.08 (-0.36, 0.19)
**Not married/cohabiting**	0.25 (-0.63, 1.12)	-0.51 (-1.04, 0.03)	-1.62 (-2.17, -1.08)	0.08 (-0.38, 0.54)
**Education**	-0.35 (-0.75, 0.06)	-0.07 (-0.28, 0.14)	0.30 (0.04, 0.56)	0.11 (-0.05, 0.27)
**Not in employment**	-0.97 (-1.84, -0.10)	-0.35 (-0.80, 0.09)	0.09 (-0.46, 0.63)	-0.44 (-0.74, -0.15)
**Current smoker**	4.46 (3.34, 5.58)	1.05 (0.37, 1.73)	-0.45 (-1.15, 0.25)	-0.11 (-0.52, 0.31)
**CAGE case**	14.87 (13.79, 15.95)	3.24 (1.93, 4.54)	-3.14 (-3.81, -2.46)	-0.09 (-0.87, 0.70)
**Physical activity**	-0.20 (-0.60, 0.20)	-0.36 (-0.60, -0.12)	-0.62 (-0.88, -0.37)	0.01 (-0.18, 0.21)
**Social network**	0.19 (0.07, 0.31)	0.13 (0.03, 0.23)	0.38 (0.30, 0.45)	-0.03 (-0.12, 0.06)
**Longstanding illness**	-0.57 (-1.28, 0.15)	-0.78 (-1.30, -0.25)	-1.93 (-2.37, -1.48)	0.23 (-0.23, 0.69)
**Known CHD**	0.47 (-0.96, 1.90)	-0.26 (-0.99, 0.46)	-0.55 (-1.45, 0.34)	-0.14 (-0.64, 0.37)

**Known diabetes**	-0.59 (-2.31, 1.13)	-0.79 (-1.69, 0.10)	-0.69 (-1.77, 0.39)	0.65 (0.03, 1.27)
**Antidepressant use**	-0.29 (-2.36, 1.78)	-2.32 (-4.18, -0.46)	-8.07 (-9.37, -6.77)	0.96 (-0.86, 2.79)
**Poor self-reported physical health**	-0.38 (-1.21, 0.45)	0.15 (-0.26, 0.57)	0.38 (-0.14, 0.91)	-1.22 (-1.52, -0.92)
**BMI**	0.20 (0.10, 0.31)	0.01 (-0.05, 0.06)	0.02 (-0.05, 0.08)	0.07 (0.03, 0.11)
**Known Stroke**	0.40 (-4.56, 5.37)	-1.46 (-4.25, 1.33)	-3.05 (-6.16, 0.06)	0.46 (-1.55, 2.47)
**Known TIA**	-2.23 (-6.27, 1.81)	1.06 (-0.97, 3.10)	-0.10 (-2.62, 2.44)	-0.18 (-1.55, 1.19)
**Cholesterol (mmol/L)**	1.58 (1.24, 1.92)	0.34 (0.13, 0.55)	-0.11 (-0.32, 0.10)	-0.06 (-0.17, 0.06)
**SBP (mmHg)**	0.08 (0.05, 0.11)	0.03 (0.01, 0.05)	0.03 (0.01, 0.05)	-0.01 (-0.02, ~0.00)
**DBP (mmHG)**	-0.08 (-0.13, -0.04)	-0.02 (-0.05, ~0.00)	-0.02 (-0.05, 0.01)	0.01 (-0.01, 0.03)
**RHR > 80 BPM**	0.64 (-0.47, 1.76)	-0.25 (-0.81, 0.30)	0.21 (-0.49, 0.91)	-0.33 (-0.70, 0.05)

SES = Socioeconomic Status, CHD = Coronary Heart Disease, BMI = Body Mass Index, TIA = Transient Ischaemic Attack, SBP = Systolic Blood Pressure, DBP = Diastolic Blood Pressure, RHR = Resting Heart Rate, BPM = Beats Per Minute.
